# ECG Measurement Uncertainty Based on Monte Carlo Approach: An Effective Analysis for a Successful Cardiac Health Monitoring System

**DOI:** 10.3390/bioengineering10010115

**Published:** 2023-01-13

**Authors:** Jackson Henrique Braga da Silva, Paulo Cesar Cortez, Senthil K. Jagatheesaperumal, Victor Hugo C. de Albuquerque

**Affiliations:** 1 Department of Teleinformatics Engineering, Federal University of Ceará, Fortaleza 60455-970, Brazil; 2 Department of Electronics and Communication Engineering, Mepco Schlenk Engineering College, Sivakasi 626005, India

**Keywords:** Measurement uncertainty, Monte Carlo method, ECG, Cardiac health

## Abstract

Measurement uncertainty is one of the widespread concepts applied in scientific works, particularly to estimate the accuracy of measurement results and to evaluate the conformity of products and processes. In this work, we propose a methodology to analyze the performance of measurement systems existing in the design phases, based on a probabilistic approach, by applying the Monte Carlo method (MCM). With this approach, it is feasible to identify the dominant contributing factors of imprecision in the evaluated system. In the design phase, this information can be used to identify where the most effective attention is required to improve the performance of equipment. This methodology was applied over a simulated electrocardiogram (ECG), for which a measurement uncertainty of the order of 3.54% of the measured value was estimated, with a confidence level of 95%. For this simulation, the ECG computational model was categorized into two modules: the preamplifier and the final stage. The outcomes of the analysis show that the preamplifier module had a greater influence on the measurement results over the final stage module, which indicates that interventions in the first module would promote more significant performance improvements in the system. Finally, it was identified that the main source of ECG measurement uncertainty is related to the measurand, focused towards the objective of better characterization of the metrological behavior of the measurements in the ECG.

## 1. Introduction

The field of medicine has considerably evolved with the help of engineering and the development of systems capable of monitoring patients and measuring their vital signs so that decisions can be made by a specialist regarding the care of that patient. In this context, the instruments used to measure and monitor a patient’s vital signs play a critical role, which requires great reliability in their measurement results. Small variations or ranges of uncertainties related to the measurement results of these instruments can lead to catastrophic effects [1]. Thus, focusing on the bioengineering perspective, measurement devices must be reliable and robust to manage such uncertainties [2].

With the current issues in the context of the analysis and development of measurement systems, one of the ways to assess the quality of measurement results is through the evaluation of the uncertainty related to the obtained results [3]. The analysis of measurement uncertainty is a task that can be applied in research and development, with several domains of knowledge acquired through theoretical, empirical, or hybrid studies [4].

Several recent works, such as [5,6,7], performed measurement uncertainty analyses to validate measurement systems and/or methods. In these works, uncertainty was used with the objective of evaluating the confidence level of the results or with the objective of comparing results obtained from different methods. In works such as [8,9], measurement uncertainty was used as a basis for decision making and conformity assessment. It is noteworthy that the work in [9] proposed a method for which the measurement uncertainty analysis showed the need to improve the metrological performance of the method with a target value for the measurement uncertainty, which is one of the recommendations in [4].

On the other hand, in many recent works such as [10,11], how the uncertainty analysis was performed or even the uncertainty of the presented results was not indicated. These works addressed various measurement methods in which, not necessarily, the measurement system was the focus. However, much attention is drawn to the fact that many other recent works, such as [12,13,14], proposed a new sensor or a new measurement system, none of which showed how the measurement uncertainty was analyzed.

Approaching it in a more specific way, the state of the art of uncertainty analysis of ECG measurement, which will be the object of study of this work, is highlighted in works such as [15], which identifies the main sources of the uncertainty in the results of ECG measurement and evaluates its influence on the QRS, SST, and QRST curves, as well as on the interpretation of these results. The work by  [16] evaluates the accuracy of its results only by the repeatability and reproducibility of an algorithm implemented to identify diseases from the digitized images of ECG curves such as the QRS, which already bring with them the uncertainties identified in works such as that of  [15]. It can be stated that in these cases, repeatability and reproducibility characterize only the uncertainty related to the process of scanning and classifying the ECG images, which must be taken into account along with all other sources of uncertainty that are present during the process of measurement and the generation of these images. In [17], the authors quantified the sources of uncertainty, using statistical techniques based on Monte Carlo, to more accurately classify cardiac arrhythmias with AI. It should be noted that in works where the uncertainty was quantified, the classification method used data that already included other uncertainties, inherited from the process of measuring the ECG signal. Several other works such as [18,19,20] used uncertainty as a parameter to evaluate the performance of the methods proposed in their respective works.

Measurement uncertainty is a parameter that makes it possible to confidently state how good a measurement method or system is or how much better it is compared with others. In works such as [21,22], the measurement uncertainty was experimentally analyzed, after interfacing the sensors with the system. However, these analyses can be carried out theoretically [3,4] and can be analyzed before designing or implementing a measurement system. Measurement uncertainty analysis can be used to show how well the behavior of a measurement system is known in the design phase and how much the performance of this system can be improved. Few works were reported on using least-square analysis for the measurement of observational uncertainties  [23] and unequally spaced non-stationary time series signals [24].

In this context, this work proposes a methodology that uses measurement uncertainty as a parameter to evaluate performance and guide actions to improve projects and the development of measurement systems. In this methodology, the Monte Carlo method (MCM) is used, the essence of which is to perform numerical simulations from a large number of repetitions and reach conclusions from the statistical analysis of the obtained responses. The proposed methodology is based on the acquired knowledge, which has been developed over time and published by the International Bureau of Weights and Measures (BIPM) in their guides. Based on such standards, MCM strategies are widely used in the literature for transmission line resistance computation [25], the assessment of truth uncertainties based on error feeds [4], and the propagation of distribution uncertainty measurement [3] applications. Furthermore, MCM was used for measuring compressive concrete strength in [26], which facilitated the analysis of robustness and sensitivity factors. In [27], MCM was used for invariance measurement for assessing the capabilities of conventional and recent measurement strategies. Additionally, the authors in [28] used MCM simulation for performing more realistic measurements in the modeling of additive manufacturing applications. It highlights the impact of the MCM approach for assessing the measurements of the lattice structures manufactured through additive techniques.

This work differs from the previous ones precisely because it uses systematic numerical simulations and uncertainty as a performance parameter during the analysis of measurement system design. The articles cited in the characterization of the state-of-the-art research on the evaluation of the uncertainties in the measurements performed with an ECG, in general, used this parameter for the interpretation and/or classification of measurement results and subsequent decision making related to the diagnosis of diseases. The detailed description of the proposed methodology of this work is elaborated in Section 2.2, which is then applied to evaluate the performance of electrocardiogram (ECG) signals. Simply put, the contribution of this work is twofold:

- Contribute to filling a small gap in the state of the art of evaluating the uncertainty of measurements performed with an ECG;
- Present a methodology capable of identifying opportunities for improvement in measurement system projects, using measurement uncertainty as a parameter.

Very recently, in  [16], the authors developed a conversion algorithm to transform image-based ECGs into digital signals. Further, in [17], an uncertainty-aware deep-learning-based predictive framework was developed for assessing the uncertainties of the model. However, none of these studies focused on uncertainty measurement in ECG signals.

Earlier, more prominent works on ECG signals were performed by authors, particularly to classify heart arrhythmia using deep learning [29], automated cardiac arrhythmia detection [30], and arrhythmia classification [31]. Further, with the support of Internet of Things (IoT) platforms, related works were reported on the classification optimization of short ECG segments [32] and atrial fibrillation recognition and detection using Artificial Intelligence of Things (AIoT).

## 2. Materials and Methods

### 2.1. Datasets

The ECG is an essential means of monitoring the cardiac activities of patients. Using standardized electrodes, carefully placed at specific points on the patient’s body, it is possible to record the heart’s electrical signals. A standard ECG uses 3, 5, or 12 electrodes [33]. With more electrodes placed over the patient’s body, more information could be acquired from the setup.

**Figure 1:**
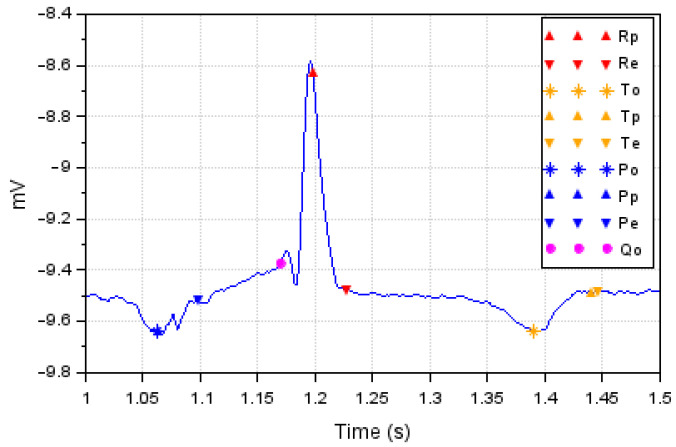
A normal ECG waveform for one cardiac cycle representing positive and negative deflection from baseline.

The ECG basically measures the electrical activities generated from the flow of blood in the heart [34]. By monitoring the heart’s electrical signals, it is possible to assess the conditions and health status of the patient. The response curves of the measured signals shown in Figure 1 indicate the normal conditions of the patients, which are obtained after the iterated processing of the signals captured from the electrodes.

**Figure 2:**
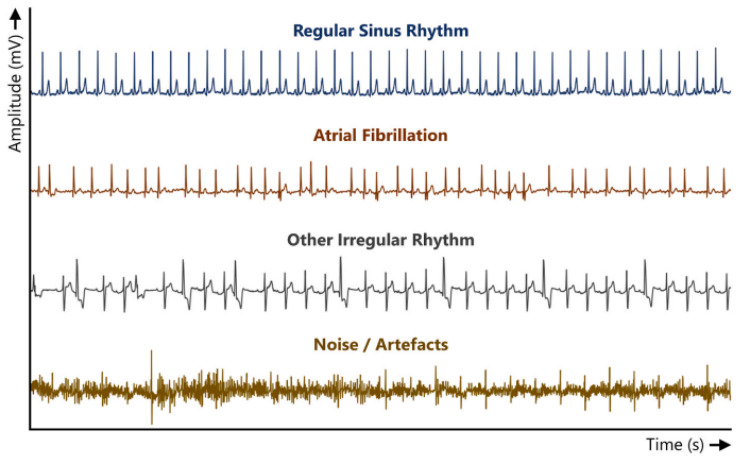
Typical ECG segments of the four different classes [35].

In Figure 2, a sample is presented of the four classes of typical ECG signals, which are postoperative telemetry data acquired from 418 patients who underwent various types of cardiac surgery [35]. These data were used to train the classification algorithm, which identifies cardiac problems based on the ECG waveform.

The noise/artifacts class, shown in Figure 2, represents those signals that cannot be interpreted by a specialist due to noise or other associated factors, e.g., patient movements or pacemaker activity [35]. There are certain crucial factors that must be taken into account when designing and using an ECG, such as frequency distortion, saturation or clipping distortion, ground loops, artifacts from large electrical transients, and interference from other electrical devices [36]. These factors are important not only for biomedical engineers but also for healthcare professionals who use this instrument in their decision making.

All sources of interference in the values indicated by an ECG generate uncertainties that may affect the interpretation of these results and, consequently, the diagnosis of diseases. Few recent works, such as [37,38,39,40], showed ways to ensure reliability when analyzing the parameters acquired by considering the ECG signals, taking into account the uncertainty of these values.

As ECG monitoring devices are widely used as a diagnostic tool, and there are several manufacturers for this instrument, performance requirements have been established by international standards over the years in order to guarantee the reliability of the values indicated by these instruments. Table 1 provides a summary of the most recent performance requirements established in the standard developed in [41].

In addition to the requirements shown in Table 1, the standard in  [41] establishes the requirements for evaluating the performance of such equipment, based on the overall system error and frequency response. Input signals should be limited in amplitude and rate of slew to ±5 mV and 125 mV/s, respectively, and should be reproduced on the output recording medium with a maximum instantaneous deviation of ±5% or ±40 microvolts (μV), whichever is greater [41].

In addition to the standard [41], which establishes minimum safety and performance requirements for ECG monitoring equipment, the International Organization of Legal Metrology (OIML) has published the international recommendation [41], which establishes requirements for the calibration and verification of the ECG monitoring system. These standards provide guidelines that can be used to identify and quantify sources of uncertainty in the measurement of ECG signals.

### 2.2. Methods

**Figure 3:**
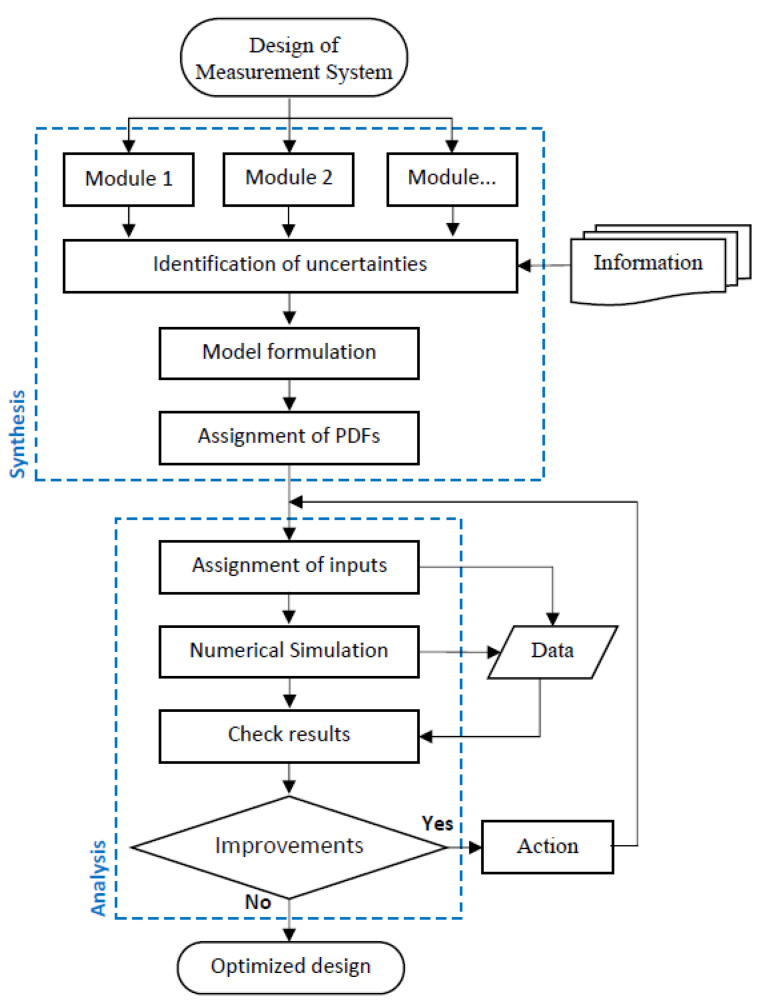
The sequence of stages involved in the synthesis and analysis phases of the proposed methodology.

The methodology proposed in this work involves the performance evaluation of a measurement system and can be applied even in the design phase. The methodology basically consists of a form of synthesis and analysis, taking into account the measurement uncertainty of the system under development. The application of this methodology is schematically presented in Figure 3.

For the performance evaluation of a measurement system, with its pre-project or initial project already elaborated, this measurement system must initially be divided into modules and, with the information gathered in the project synthesis, the input quantities and primary sources of measurement uncertainty must be identified.

It is necessary to know how these modules are interconnected, and how they behave individually and together. With this knowledge, it is possible to determine a mathematical model for the system, capable of characterizing the metrological behavior of the complete system, as well as the behavior of each module individually. Guidelines for the mathematical modeling of a measurement system can be found in [4].

As the analysis is performed using statistical tools, it is necessary to assign each of the sources of uncertainty a probability density function (PDF) that characterizes its random behavior [25].

The analysis phase highlighted in Figure 3 presents the iterative process where the various ranges and other necessary parameters are assigned to the input quantities. Following this process, the MCM is applied through the numerical simulation of the previously defined mathematical model, and the outputs are analyzed in comparison with the desired performance of the system.

In addition to this methodology, proposing the use of measurement uncertainty as a parameter to evaluate the performance of a measurement system, the application of the MCM also stands out for numerical simulations using a probabilistic approach, which can be implemented in software for mathematical computation, as shown in Algorithm 1.
**Algorithm 1** MCM implementation.   $\;\;\;\;X\left[x_{1},x_{2},x_{3},\ldots ,x_{n}\right];U\left[u_{1},u_{2},u_{3},\ldots ,u_{n}\right]$$M\leftarrow c_{1}$ //Initialize M (number of iterations)A[n:M] // The array A is declared$A\left(1,1:M\right)\leftarrow f\left(M,x_{1},u_{1},pdf\right)$ //Assigns random number with proper PDF$A\left(2,1:M\right)\leftarrow f\left(M,x_{2},u_{2},pdf\right)$$A\left(3,1:M\right)\leftarrow f\left(M,x_{3},u_{3},pdf\right)$⋮$A\left(n,1:M\right)\leftarrow f\left(M,x_{n},u_{n},pdf\right)$   Y[n+1:M+2] //The array Y is declared Y(n+1,1:M)←g(A) //Function g defines the mathematical modelY(n+1,M+1)←average(Y(n+1,1:M))A(n+1,M+2)←standardDeviation(Y(n+1,1:M))   B[n:M]←h(n,X) //The array B is declared with n lines constants    for i=1 to *n* Z[1:M]←B(i:M)B(i:M)←A(i:M)Y(i,1:M)←g(B)Y(i,M+1)←average(Y(i,1:M))Y(i,M+2)←standardDeviation(Y(i,1:M))B(i:M)←Z

This algorithm requires coherent values as input variables for the quantities under analysis and estimates the measurement uncertainties associated with each of the input parameters. Its output is a data vector containing the values of the output quantity considering the influence of each measurement uncertainty source individually, as well as considering the influence of all uncertainty sources acting concurrently.

The *M* parameter is the minimum number of simulations recommended for the MCM application. This number depends on the desired confidence level *p* (or coverage probability) for the application so the higher the desired confidence level, the greater the *M* should be and, consequently, the greater the computational effort required for simulation. *M* can be determined by Equation (1).

(1) $$M=\frac{1}{\left(1-p\right)}\cdot 10^{4}$$

The number of input variables is represented by *n*, and function *f*, in Algorithm 1, is used to generate random numbers according to the PDF suitable for the behavior of the measurement uncertainty associated with the input variable. The measurement uncertainty expression guide [25] provides valid recommendations for PDF assignments.

The looping statement in Algorithm 1 is implemented to evaluate the influence on the output, based on the uncertainty source acting individually. However, these looping statements can be modified to assess the influence of a group of uncertainty sources, which would characterize the behavior of a system module.

It is worth noting that the mathematical model implemented through numerical simulation helps to gain awareness of the metrological behavior of the system as a whole, as well as of each module individually. Through this analysis, the relative performance of each module against the performance of the complete system could be evaluated. This analysis is very convenient to identify which action will promote a significant improvement in the performance of the system, as well as to evaluate the costs for such improvement. Thus, an optimized design can be achieved by considering the best cost–benefit ratio.

The methodology proposed in this work was applied to evaluate the performance of ECG signal measurement. In Section 2.1, the formation of the knowledge/information base is presented, which basically comprises the description of the measurement process through the parameters and metrological requirements that are necessary to characterize and delimit the system under development. The synthesis and analysis phases of the proposed methodology are presented in Section 3.

## 3. Results

**Figure 4:**
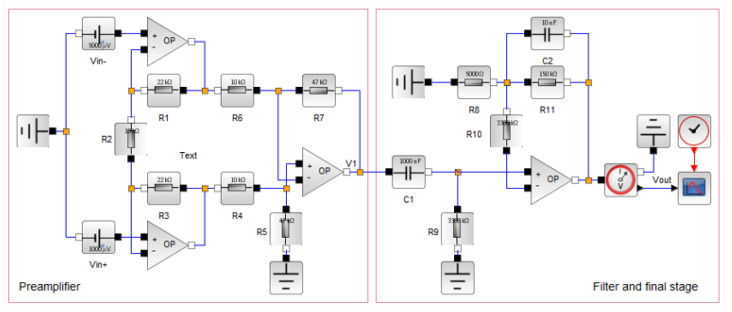
A simplified ECG system with preamplification and filter stages.

The application of the proposed methodology began with the collection of information and the clear definition of the measurement system, with the identification of modules and other fundamental parts for its proper functioning. In this application, the high input impedance electrical circuit module is presented in Figure 4, which was divided into two modules. The first half was the preamplifier phase, where the first stage of amplification of the input signal occurred. In the second half of the module, the signal was filtered and passed through the second amplification stage.

### 3.1. Formulation of the Model

The ECG monitoring system design, shown in Figure 4, was modeled using the Xcos tool from Scilab version 6.1.1, a free open-source cross-platform numerical computational tool. Considering the few idealizations for the circuit represented in Figure 4, we have $R_{1}=R_{3}$; $R_{4}=R_{6}$; $R_{5}=R_{7}$, and $R_{9}=R_{10}$, for which the transfer function can be formulated as shown in Equation (2), for the preamplifier, and in Equation (3), for the final stage:

(2) $$v_{1}=\left(1+\frac{2R_{1}}{R_{2}}\right)\frac{R_{5}}{R_{4}}\left(v_{in+}-v_{in-}\right)$$

(3) $$v_{out}=\left(1+\frac{R_{11}}{R_{8}}\right)\left(v_{1}\right)$$

From Equations (4) and (5), we could estimate the cut-off frequencies of the first and second modules, respectively, which are responsible for attenuating the effect of noise in the input signal.

(4) $$f_{1}=\frac{1}{2\pi C_{1}R_{9}}$$

(5) $$f_{2}=\frac{1}{2\pi C_{2}R_{11}}$$

With these equations, it is possible to evaluate the behavior of each module, in isolation and of the system as a whole. It is also possible to evaluate the contribution of each element of this circuit to estimate the accuracy of the system. Moreover, it guarantees the possibility to identify exactly where to act, substituting an element or improving the performance of a specific module and, consequently, of the measurement system under development.

### 3.2. PDF Assignment

For each of the uncertainty sources, which were considered to be significant in the previous analysis, quantities were assigned, their average value was determined (μ), and their range of variation was characterized by the standard deviation (σ) or between an interval of (a,b). In addition to the quantities, the assigned PDFs characterized their random behavior. Table 2 presents the quantities and the PDF of the uncertainty sources considered for analysis in this article.

The parameters presented in Table 2 were categorized into three groups of factors, with the aim of better organizing the knowledge about the metrological behavior of ECG signals. The first group gathered the factors related to the measurement, that is, the electrical signals, which were the factors not completely under the control of whoever develops the measurement system. The second group brought together the factors related to the measurement system, which were factors internal to the system that could be analyzed to identify opportunities for improvement in the system. Finally, the third group gathered the external factors, which were the factors related to the environment, where the measurements were carried out. The factors related to the environment were not the focus of the application but must be treated with due attention.

**Figure 5:**
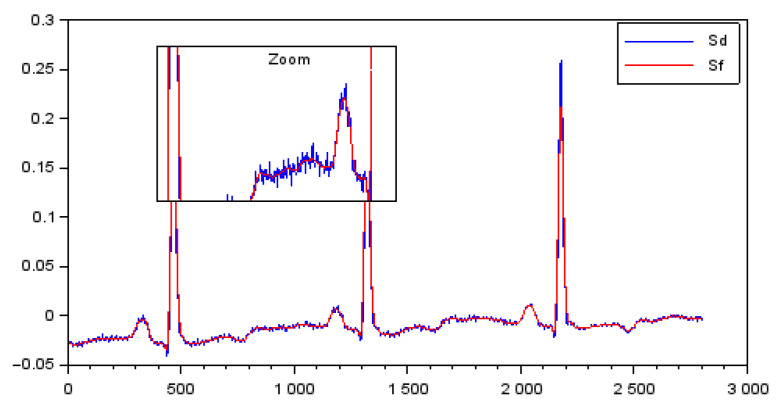
Detrended signal (Sd) and filtered signal (Sf).

In this work, all external interference was considered for analysis in the form of noise inputs pertaining to the measurement signal. The analysis was carried out through the Cardiovascular Wave Analysis module of the Scilab software. This module provides ECG data files (open-access databases) that were used in the simulations performed in this work. In Figure 5, the signal generated by this tool is depicted, from which the parameters related to the baseline and noise of the signal were obtained.

In the proposed methodology, the MCM was used to analyze the sources of uncertainty, for which inferences were estimated through numerical iterations. Moreover, the method is also recommended for situations in which the linearization of the mathematical model of measurement provides an inadequate representation, or the PDF of the output quantity significantly deviates from a Gaussian distribution or a *t*-distribution [25].

The essence of MCM is to perform numerical simulations from a large number of repetitions and to obtain conclusions about the phenomenon under study from the statistical analysis of the responses obtained. The MCM in this work was carried out with Algorithm 1, following the prescribed measurement guidelines [25].

For the implementation of the MCM, normal, rectangular, and U-shaped PDFs were often used to achieve the desired characteristics of the system under test. The PDFs used to generate the sample values were implemented in the Scilab software tool. For this implementation, $M=2\times 10^{5}$ samples were used in order to obtain results, with a confidence level of 95%.

**Figure 6:**
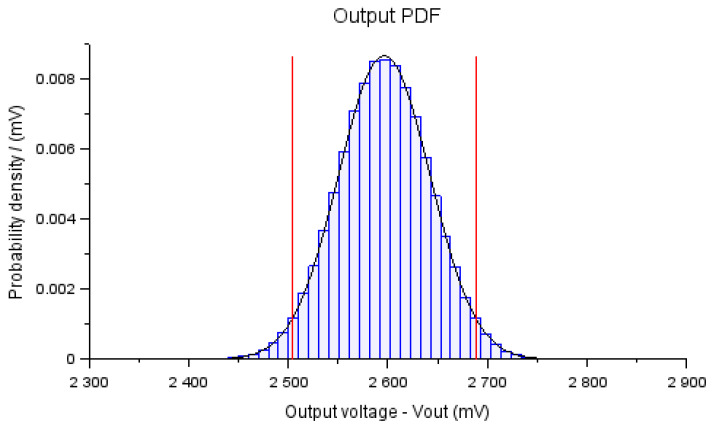
PDF for $V_{out}$ obtained using the MCM for the approximate model (3) using the information summarized in Table 2.

As an initial response, output data were obtained with a normal probability distribution, providing a mean of 2596 mV, and a standard deviation of 57 mV, as shown in Figure 6. The measurement uncertainty, calculated for a coverage probability of 95%, was ±112 mV, which corresponded to 4.32% of the mean value.

The previous results refer to the simulation in which all uncertainty sources acted simultaneously. However, this simulation strategy can also be applied by varying only one or a set of uncertainty sources at a time, to assess their level of influence on the results.

**Figure 7:**
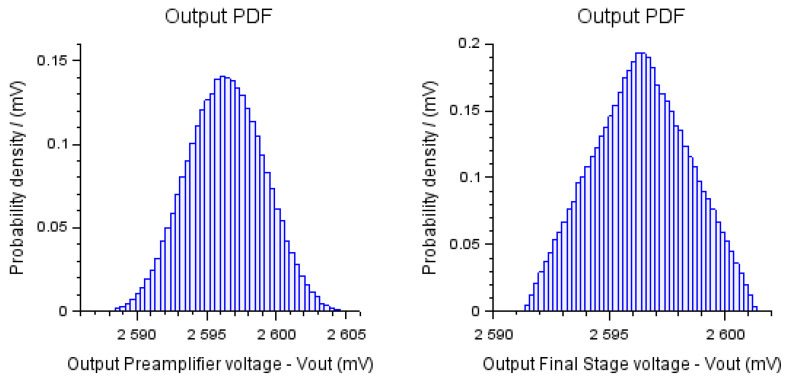
PDF for preamplifier and final stage.

Figure 7 shows the PDFs obtained with the application of the MCM for the two modules of the measurement system under study, and they were analyzed separately. It is noteworthy that the probability function of the first module followed a normal curve, and the second module formed a triangular curve. This fact highlights the importance of using MCM in this methodology since traditional analytical methods, as observed in [3], assume that the outputs are characterized by a normal probability distribution curve.

The use of MCM guarantees greater assurance in the results obtained with the application of the methodology proposed in this work, since this method allows the propagation of uncertainty in modules, in addition to the propagation of the PDFs [25].

In Table 3, the results of the simulation of the sources of uncertainty considered significant in this work are tabulated based on the analysis performed individually as well as in blocks.

In the initial analysis, the use of precision resistors of 1% was considered in the electrical circuit of the setup (Table 2). By considering the use of high-precision resistors of 0.1% of the nominal value only in the preamplifier module, an output with a mean of 2596 mV, a standard deviation of 50 mV, and an uncertainty of 99 mV was obtained, which corresponded to 3.80% of the average value. It is evident from this observation that, in terms of the average value, the contribution of the preamplifier module dropped from 2.07% to 0.21%.

Considering the use of resistors with an accuracy of 0.1% in the entire experimental setup shown in Figure 4, an uncertainty of 90 mV was achieved, which corresponded to 3.47% of the average value. This indicated a 0.85% improvement in the accuracy of the ECG signal under analysis. In Table 4, the simulation results are presented considering the implementation, with the suggested improvement actions.

A comparison of the quantitative results of Table 4 with the results presented in Table 3 highlights the potential of the methodology proposed in this work to identify and direct improvement actions in measurement system projects, which, in turn, can be analyzed through computer simulations before their respective implementations.

### 3.3. Validation and Comparisons with the Literature

In terms of evaluating measurement uncertainty, the most widespread method in the literature is the analytical method published in ISO-GUM, cited in works such as [3,4,25]. In these studies, basically, a combined standard uncertainty is calculated at approximately 68% confidence level, using the expression:

(6) $$u\left(Y\right)=\sqrt[{}]{{\left(\frac{\partial y}{\partial X_{1}}u\left(X_{1}\right)\right)}^{2}+{\left(\frac{\partial y}{\partial X_{2}}u\left(X_{2}\right)\right)}^{2}+\ldots +{\left(\frac{\partial y}{\partial X_{n}}u\left(X_{n}\right)\right)}^{2}}$$

where u(Y) is the combined standard uncertainty of the output quantity, and $u(X_{i})$ is the standard uncertainty assigned to the i-th input quantity being combined. To calculate the expanded uncertainty for a confidence level of 95%, Equation (7) is used:

(7) $$U_{95}=k\cdot u\left(Y\right)$$

where k=1.96 for a confidence level of 95%, considering the effective degrees of freedom tending to infinity.

Applying this method to the problem in question and taking the data from Table 2 as inputs, an uncertainty of ±161 mV was found, which corresponded to 6.21% of the measured value. It is noteworthy that, for the same parameters and input values, the value found with the methodology proposed in this work was 4.32% of the measured value.

Comparing the result obtained by applying the methodology presented in this work with the result of applying the methodology used in the literature on the evaluation of measurement uncertainty, it is highlighted that the methodology proposed in this work presented more precise results.

## 4. Discussion

As shown in Table 3, baseline variations and noise interference had a negligible influence on the obtained results. From these results, it is evident that the most significant uncertainty was associated with variations in the input signal.

There are several causes for input signal variations as the most significant source of uncertainty, such as the placement of sensors on the patient’s body, patient movements during measurements, electromagnetic interference from other equipment, and other sources of uncertainty that are not under the control of who designs the measurement system.

It is noteworthy that the second most significant source of uncertainty was associated with the preamplifier module. From the estimated observations on the uncertainty information, the designer can assess how much the measurement system uncertainty can be reduced by acting on a specific module in the system.

As previously stated in the aforementioned discussion, the source with the greatest contribution of uncertainty was related to the input signal, which in turn was related to the measurand. However, it is noteworthy that the identified improvement actions promoted significant reductions in the contributions of the analyzed modules. To promote further improvements, it would be recommended to act smartly on system parameters to improve the stability of the baseline or reduce the effect of noise on the measured signal.

**Figure 8:**
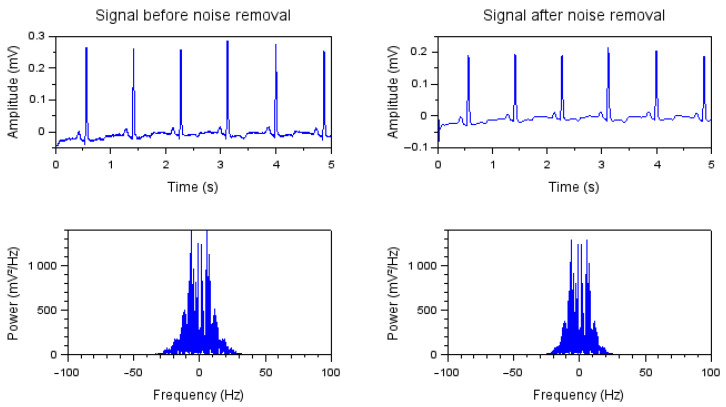
ECG signals power as a function of frequency before and after noise removal.

In Figure 8, the power spectrum of the noisy signal is plotted centered at the zero frequency before and after noise removal. The power amplitude is represented as the squared magnitude of a signal’s Fourier transform, normalized by the number of frequency samples. If the input signal noise, as well as the signal itself, is of low frequency (below 50 Hz before the filter), it would be challenging to remove the noise without significantly affecting the signal of interest. With the chosen filter applied in this work, it was possible to remove noise with a frequency above 30 Hz, as can be observed in Figure 8.

**Figure 9:**
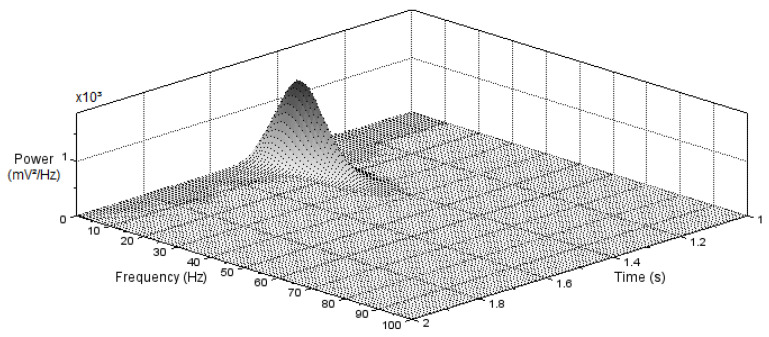
Time–frequency spectrogram after noise removal.

In the spectrogram of the filtered signal, shown in Figure 9, which covers the time interval of 1 to 2 s, it is possible to notice that the remaining noise and a good part of the signal of interest were of low power and practically constant over the measurement period. The analysis of Figure 8 and Figure 9 reveals that particular attention must be paid when applying filters so that the significant data of the ECG signal of interest are not lost.

In order to improve the performance of the ECG acting on the source of uncertainty related to the input signal, in addition to the use of signal-processing techniques and noise filtering, it is necessary to carry out investigations taking into account the measurement procedure and the functioning mode of the used sensor. In future works, procedures that can measure the electrical activity of the heart, or even other signals, can be investigated so that the results are not so influenced by the positioning of the sensors and the movement of the patients. Likewise, future investigations can be carried out with the aim of identifying sensors that are not as susceptible to noise from the environment.

As regards the limitations of the methodology proposed in this work, it should be noted that it was tested with problems in the time domain; therefore, for applications in the frequency domain, adjustments in the proposed algorithm are necessary. It should also be noted that the successful implementation of this methodology is strongly limited by the ability of the mathematical model to describe the metrological behavior of the elements and/or modules that constitute the measurement system under analysis.

## 5. Conclusions

The methodology presented in this work demonstrates the probabilistic uncertainty from the measurement system, with the measurements and analysis performed on an ECG monitoring system. The methodology uses a probabilistic approach for the evaluation of measurement uncertainty, through the application of the MCM, to evaluate the performance of signals measured from an ECG monitoring system. With the performed analysis, it was possible to reach the desired situation, for which the ECG measurement uncertainty would be 3.47% in relation to the measurement result, subjected to a confidence level of 95%. With this analysis, it was also possible to identify strategic points where actions can be taken to further improve the accuracy of the measurement system, such as actions to improve baseline stability or actions to reduce the effect of noise. It is noteworthy that the application of this methodology revealed that the sources of uncertainty related to the input signal, directly related to the measurand, was 3.36% of the measured value, which was almost the measurement uncertainty of the ECG itself. Thus, studies can be carried out with the objective of better investigating the behavior of the measurement system and, consequently, improving the measurement process, as well as increasing the reliability of the results. It is concluded that a more detailed and reliable understanding of the behavior of a measurement system, as well as the individual behavior of an element or a module of that system, makes it possible to act more efficiently to improve the method’s performance.

## Figures and Tables

**Table 1:** Requirement of ECG monitoring devices and their description [41].

Requirement Description	Min/Max	Units	Value
Operating conditions:			
Line voltage	Range	V RMS	104 to 1127
Frequency	Range	Hz	60±1
Temperature	Range	${}^{\circ }$C	25±10
Relative humidity	Range	%	50±20
Atmospheric pressure	Range	kPa	70 to 106
Input Dynamic Range:			
Range of linear operations of input signal	Min	mV	±5
Allowed variation of amplitude with dc offset	Max	%	±5
Gain control, accuracy, and stability:			
Gain error	Max	%	5
Gain change rate/min	Max	%/min	±0.33
Total gain change/h	Max	%	±3
Time base selection and accuracy:			
Time base error	Max	%	±5
Output display:			
Error of rulings	Max	%	±2
Time marker error	Max	%	±2
Accuracy of input signal reproduction:			
Overall error for signals	Max	%	±5
Error in lead weighting factors	Max	%	5
Hysteresis after 15 mm deflection from baseline	Max	mm	0.5
Standardizing voltage:			
Amplitude error	Max	%	±5
System noise:			
Multichannel crosstalk	Max	%	2
Baseline stability:			
Baseline drift rate RTI	Max	μV/s	10
Total baseline drift RTI (2 min period)	Max	μV	500

**Table 2:** The input quantities and their PDFs assigned on the basis of available information.

Quantity	PDF	Parameters				
		μ	σ	a	b	Unit
Measurand:						
$v_{in+}$	N(μ,σ)	0.30	0.04			mV
$v_{in-}$	N(μ,σ)	0.00	0.04			mV
Baseline	N(μ,σ)	3.00	0.01			mV
Measuring system:						
$R_{1}$	R(a,b)	22.00		21.78	22.22	kΩ
$R_{2}$	R(a,b)	10.00		9.90	10.10	kΩ
$R_{4}$	R(a,b)	10.00		9.90	10.10	kΩ
$R_{5}$	R(a,b)	47.00		46.53	47.47	kΩ
$R_{8}$	R(a,b)	5.00		4.95	5.05	kΩ
$R_{9}$	R(a,b)	3.30		2.27	3.33	MΩ
$R_{11}$	R(a,b)	150.00		148.50	151.50	kΩ
$C_{1}$	U(a,b)	1.00		0.99	1.01	μF
$C_{2}$	U(a,b)	10.00		9.90	10.10	nF
Environment:						
Noise	N(μ,σ)	0.00	0.01			mV

**Table 3:** Individual or block simulation of uncertainty sources.

Source of Uncertainty	Vout (mV)			$U_{95}\left(\%\right)$
	μ	σ	$U_{95}$	
Measurand:				
$v_{in}$	2596	44	87	3.36
Baseline	2596	8	15	0.59
Measuring system:				
Preamplifier	2596	27	54	2.07
Final stage	2596	20	39	1.55
Environment:				
Noise	2596	8	15	0.59

**Table 4:** Individual or block simulation of uncertainty sources after design improvements.

Source of Uncertainty	Vout (mV)			$U_{95}\left(\%\right)$
	μ	σ	$U_{95}$	
Measurand:				
$v_{in}$	2596	45	87	3.36
Baseline	2596	8	15	0.59
Measuring system:				
Preamplifier	2596	3	5	0.21
Final stage	2596	2	4	0.15
Environment:				
Noise	2596	8	15	0.59

## Data Availability

Not applicable.
